# Type IV choledochal cyst with polycystic kidney disease: a case report

**DOI:** 10.1186/s12876-020-01445-2

**Published:** 2020-09-21

**Authors:** Yuxin He, Zhuwen Yu, Weichang Chen

**Affiliations:** Department of Gastroenterology, The First Affiliated Hospital of Soochow University, Soochow University, 188 Shizi Road, Suzhou, Jiangsu Province China

**Keywords:** Choledochal cyst, Polycystic kidney disease, Caroli disease

## Abstract

**Background:**

Choledochal cysts are divided into 5 types. Physicians believe that Caroli disease (which refers to type V biliary cysts) is a special type of biliary cyst caused by a mutation in the *PKHD1* gene and is associated with autosomal recessive polycystic kidney disease (ARPKD). There is currently no clear association between other types of choledochal cysts and polycystic kidney disease.

**Case presentation:**

We report a 65-year-old male patient with jaundice, decreased appetite, and itchy skin. His biochemistry test results indicated obstructive jaundice disease. Cross-sectional imaging showed a type IVA choledochal cyst accompanied by autosomal dominant polycystic kidney disease (ADPKD). Due to economic difficulties, the patient achieved percutaneous transhepatic cholangial drainage (PTCD) instead of surgery.

**Conclusion:**

To our knowledge, this is the second case report of the coexistence of type IVA choledochal cysts and ADPKD. We conclude that it is vital to be aware that the above condition is a possibility. This case report will aid earlier diagnosis and management and possibly prevent further damage to liver and kidney function.

## Background

Choledochal cysts are cystic dilations that may occur in any part of the bile duct between the liver and the duodenum. Alonso-lej first classified choledochal cysts in 1959 [1], and Todani expanded the classification system in 1977 by dividing choledochal cysts into 5 types [2]. Type I cysts are subclassified into 3 types. Type IA cysts are marked cystic dilations of the entire extrahepatic biliary tree that do not involve the intrahepatic ducts. Type IB cysts are focal, segmental dilations of the extrahepatic bile duct. Type IC cysts are smooth fusiform dilations of the entire extrahepatic bile duct. Type II cysts are discrete diverticula of the extrahepatic duct with narrow stalk connections to the common bile duct. Type III cysts are choledochoceles that are located in the duodenal wall. Type IVA cysts are multiple intrahepatic and extrahepatic dilations. Intrahepatic duct dilation can be cystic, fusiform, or irregular. Type IVB cysts are multiple dilatations that are confined only to the extrahepatic bile duct. Type V cysts, which are also called Caroli disease, show single or multiple dilatations of the intrahepatic bile duct.

The aetiology of choledochal cysts is complex. At present, there are several assumed causes, including genetic factors, abnormal pancreaticobiliary confluence (pancreaticobiliary maljunction, PBM), gastrointestinal neuroendocrine factors, and abnormal proliferation of the bile duct epithelium. Caroli disease is an autosomal recessive genetic disease that is caused by a mutation in the *PKHD1* gene located on chromosome 6p12 [3]; usually, it is considered to be associated with autosomal recessive polycystic kidney disease (ARPKD). The role of congenital factors in the pathogenesis of choledochal cysts aside from those associated with Caroli disease is not yet clear.

## Case presentation

A 65-year-old male presented with jaundice for half a month, which was accompanied by decreased appetite and itchy skin without abdominal pain. The patient had white stool and yellow urine. Upon physical examination, this patient showed an anaemic appearance. There was no detectable hepatosplenomegaly. His abdomen was unremarkable, and there was no tenderness throughout the abdomen. Murphy’s sign was absent on physical examination. He had never undergone any imaging examination previously. Except for a history of hypertension for 20 years, he had no other specific diseases.

The routine blood examination indicated anaemia (haemoglobin, 104 g/L), and the white cell count and platelets were within the normal limits. The biochemistry test results were as follows: albumin 32 g/L, alkaline phosphatise 395.1 U/L, gamma glutamyl transferase 100.4 U/L, alanine aminotransferase 25.3 U/L, aspartate aminotransferase 30.3 U/L, bilirubin 331.3 μmol/L, direct bilirubin 243 μmol/L, indirect bilirubin 88.30 μmol/L, creatinine 119.7 μmol/L, blood urea nitrogen 11.4 mmol/L, eGFR 54.51 ml/(min/1.73 m^2^). The prothrombin time and activated partial thromboplastin time were normal (11.7 and 29.8 s, respectively).

**Fig. 1:**
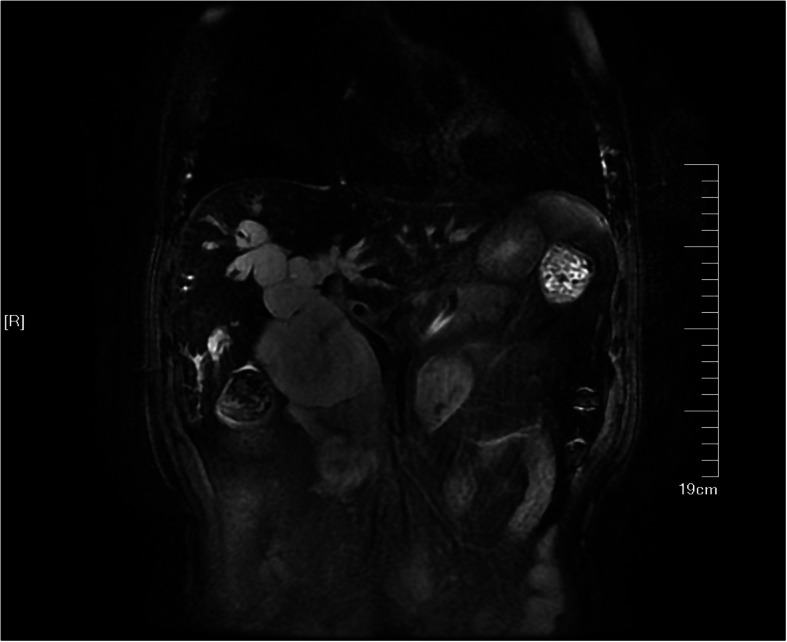
MRCP shows cystic dilatation of the intra-hepatic ducts and extrahepatic bile ducts, referring to type IVA choledochal cyst

Magnetic resonance imaging (MRI) and magnetic resonance cholangiopancreatography (MRCP) showed cystic dilatation of the intrahepatic ducts and extrahepatic bile ducts (Fig. 1). The observation of cystic dilatation of the intrahepatic ducts and extrahepatic bile ducts led to the diagnosis of choledochal cysts (type IVA). The diameters of the left kidney are as follow: 138.5 mm in length, 64.1 mm in width, and 57.9 mm in thickness. Length, width and thickness of the right kidney are 154.7 mm, 78.6 mm, and 55.8 mm, respectively.

**Fig. 2:**
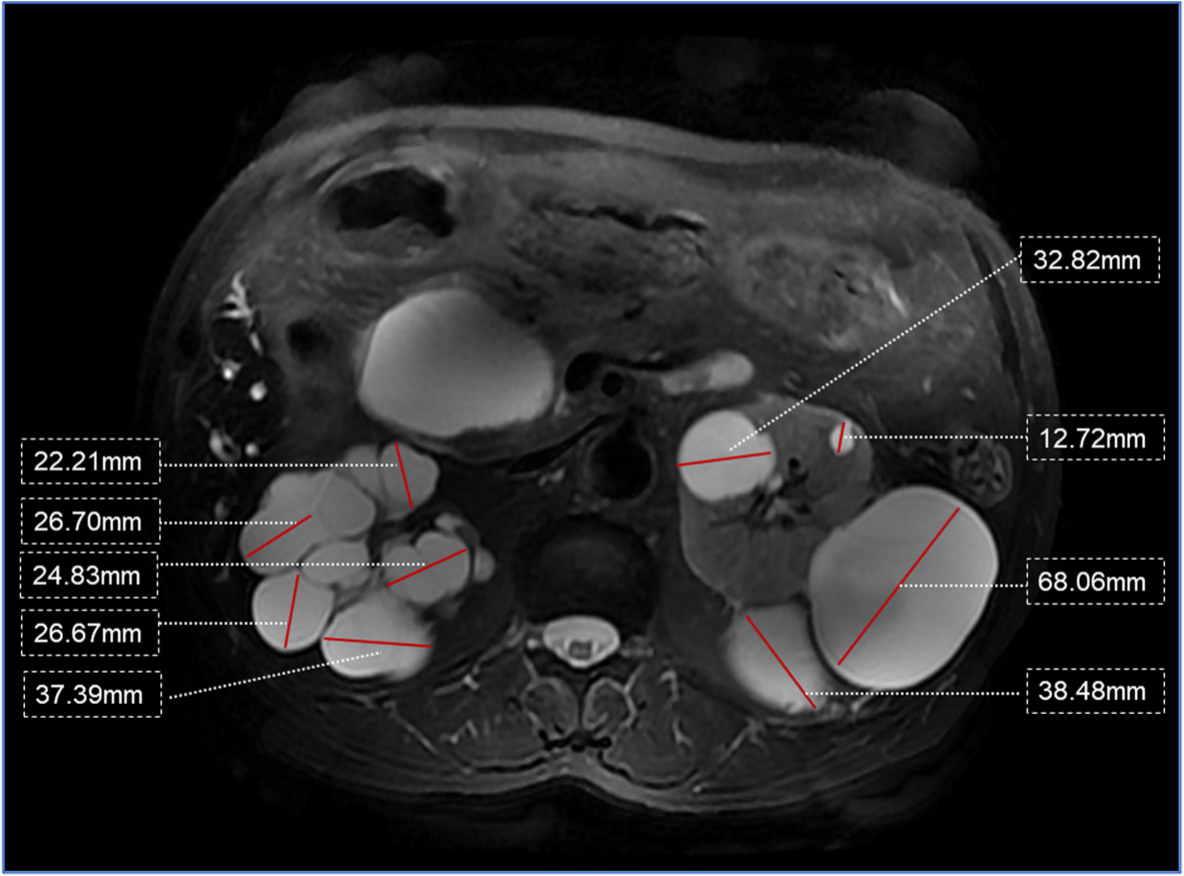
A renal MRI with 4 or more cysts with 1 cm or more in each kidney leads to the diagnosis of ADPKD

In addition, MRI showed evidence of polycystic kidney disease. We proposed that the patient’s family members undergo a kidney imaging examination. A renal ultrasound showed 3 cysts in the patient’s daughter, who was 39 years old.. According to the ultrasound-based criteria, the presence of 3 or more uni-or bilateral renal cysts is sufficient to establish the diagnosis of autosomal dominant polycystic kidney disease (ADPKD) in individuals with a positive family history of this disease younger than 40 years old (see Supplementary Materials) [4]. A renal MRI showing 4 or more cysts with at least 1 cm in size in each kidney, in turn, is acceptable for the diagnosis of ADPKD in patients aged 60 years or older with a positive family history of ADPKD, criteria that were fulfilled by our 65-year-old patient. Lastly, the presence of 2 or more cysts with at least 1 cm in each kidney on MRI in individuals within the age range of 40–59 years and with a positive family history is acceptable to make the diagnosis of ADPKD, diagnostic criteria that were fulfilled by the 42-year-old patient’s son, who also presented hepatic cysts (see Supplementary Materials). Similarly, the patient met the MRI-based diagnostic criteria (Fig. 2). This analysis provided evidence of an inherited disease, revealing a family with ADPKD.

Due to economic difficulties, the patient decided not to undergo surgery. Later, he achieved percutaneous transhepatic cholangial drainage (PTCD). His postoperative biochemistry test results were improved: albumin 40.2 g/L, alkaline phosphatise 332.9 U/L (normal < 120), gamma glutamyl transferase 75.8 U/L, alanine aminotransferase 36.9 U/L, aspartate aminotransferase 35.5 U/L, bilirubin 249.49 μmol/L, direct bilirubin 208.87 μmol/L, indirect bilirubin 40.62 μmol/L, creatinine 100.3 μmol/L, and blood urea nitrogen 9.0 mmol/L.

The patient has been followed-up for 7 months, and there have been no serious events as of January 2020. His bilirubin did not increase further; in addition, his creatinine and blood urea nitrogen were normalized.

## Discussion and conclusions

Existing knowledge of the relationship between choledochal cysts and polycystic kidney disease should be expanded. Physicians believe that Caroli disease is caused by a special type of biliary cyst caused by mutations in the *PKHD1* gene. It is associated with autosomal recessive polycystic kidney disease (ARPKD). There is currently no clear association between other types of choledochal cysts and polycystic kidney disease.

ADPKD is most commonly caused by mutations in PKD1 or PKD2 [5]. PKD1, which is located on chromosome 16 (16p13.3) encodes polycystin-1 (PC1) [6]. PKD2, which is located on chromosome 4 (4q21) encodes polycystin-2 (PC2) [7]. Both PC1 and PC2 are found in the primary cilia. Loss of PC1 or PC2 is associated with low intracellular calcium concentrations, leading to increased activity of adenyl cyclase, reduced phosphodiesterase 1 activity, excessive cyclic AMP (cAMP) concentrations, and cystogenesis through the activation of proliferation and secretion pathways [8]. Heterozygous mutations in *GANAB* were found in a minority of families with autosomal dominant polycystic kidney or liver disease. *GANAB* encodes the α subunit of glucosidase II, an enzyme involved in N-linked glycosylation, which is a key control process that governs folding, maturation, and trafficking of membrane and secreted proteins [9]. Notably, the β subunit of this enzyme is encoded by *PRKCSH*, one of the main genes involved in autosomal dominant polycystic liver disease [10].

Choledochal cysts are characterized by progressive bile duct dilatation and/or cyst development. Babbitt’s theory [11] has gained much popularity and makes the assumption that biliary cysts are caused by abnormal pancreaticobiliary junctions (APBJs). The pancreatic duct and the common bile duct meet at a site that lies outside of the area of influence of the papillary sphincter, thus forming a long common channel, and leading to the reciprocal reflux of pancreatic juices and bile. Usually, the intraductal pressure in the pancreatic duct is higher than that in the bile duct [12], so the belief that the pancreatic juice returns to the biliary tract is undisputed. The active pancreatic enzymes cause inflammation and deterioration of the biliary duct wall, leading to dilatation. Moreover, higher pressure in the pancreatic duct can further enlarge a weak wall cyst. There is another theory that states that biliary cysts are congenital in nature. The biliary system develops from the hepatic diverticulum which originates from the foregut. In approximately the 4th week of gestation, APBJ develops due to dysplasia of the ventral pancreas. Usually, the cranial pancreatic anlagen duct disappears; if it remains, it may cause APBJ. If ventral pancreatic atypical dysplasia leads to occlusion of the common bile duct at the same site, biliary cysts will occur [13].

Caroli disease is considered a possible concomitant disease in patients with autosomal recessive polycystic kidney diseases (ARPKD). The gene underlying ARPKD is called *PKHD1* and maps to chromosome 6 (6p21-p12). *PKHD1* encodes a large protein, named fibrocystin [14]. Fibrocystin shares structural features with the hepatocyte growth factor receptor and takes part in the regulation of cell proliferation and cellular adhesion and repulsion. The disruption of *PKHD1* results in abnormalities in ciliary morphology and biliary cystogenesis [15].

To our knowledge, this is the second case reporting the coexistence of type IVA choledochal cysts and ADPKD; the first case reported the coexistence of type IV A and type III choledochal cysts with ADPKD [16]. We hope to attract the attention of researchers. Possibly, there are new theories about biliary cysts and polycystic kidney disease. Acknowledgement of the possible coexistence of type IVA choledochal cysts and ADPKD will lead to earlier diagnosis and management and possibly prevent further damage to liver and kidney function.

Patients with type IVA biliary dilatation should undergo cholecystectomy, resection of the involved liver segment and extrahepatic bile duct and choledochojejunostomy. Our patient chose not to undergo surgery due to economic difficulties. We acknowledge this as a limitation.

Urribarri [17] found that choledochal cysts are associated with matrix metalloproteases (MMP) hyperactivity in cholangiocytes resulting from autocrine/paracrine stimulation by IL-6 and IL-8. Inhibition of MMP hyperactivity offers a potential therapeutic strategy. Onori et al. [18] found that follicle-stimulating hormone promotes biliary growth by activating the cAMP/ERK signalling pathway. This kind of disease is also expected to be treated non-surgically in the future, providing more choices for physicians and patients.

## Supplementary information

**Additional file 1.** Ultrasound of the patient’s daughter (39 years old). Renal MRI of the patient’s son (42 years old).

## Data Availability

The datasets used and/or analyzed during the current study are available from the corresponding author on a reasonable request.

## Abbreviations

ADPKD: Autosomal Dominant Polycystic Kidney Disease
APBJ: Abnormal pancreaticobiliary junction
ARPKD: Autosomal Recessive Polycystic Kidney Disease
MMPs: Matrix metalloproteases
MRCP: Magnetic resonance cholangiopancreatography
MRI: Magnetic resonance imaging
PTCD: Percutaneous transhepatic cholangial drainage
PBM: Pancreaticobiliary maljunction
