# Supplementary figures and images for: Type IV choledochal cyst with polycystic kidney disease: a case report
Source: BMC Gastroenterol. 2020 Sep 21;20:306. doi: 10.1186/s12876-020-01445-2 (PMC7507809; doi:10.1186/s12876-020-01445-2)

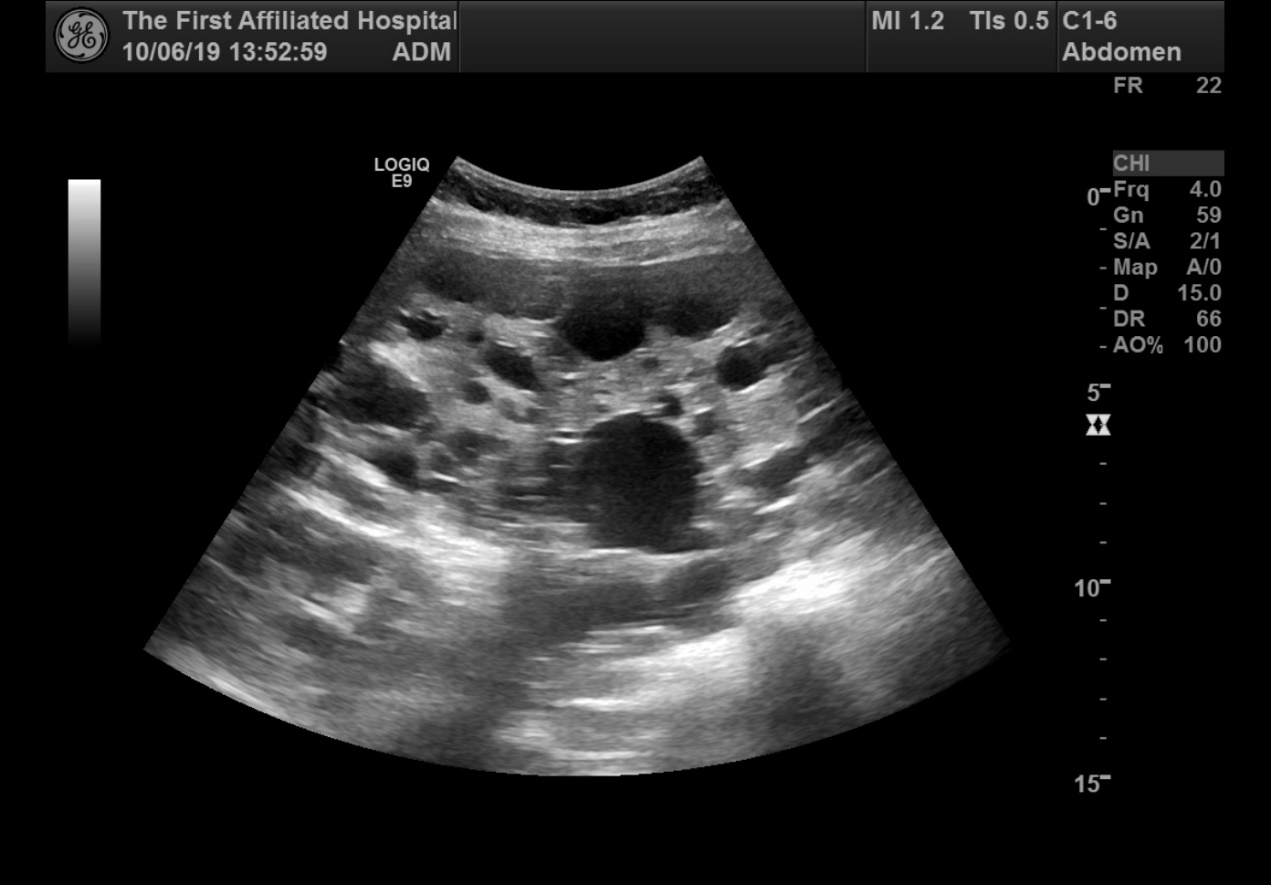


Ultrasound of the patient’s daughter (39 years old)


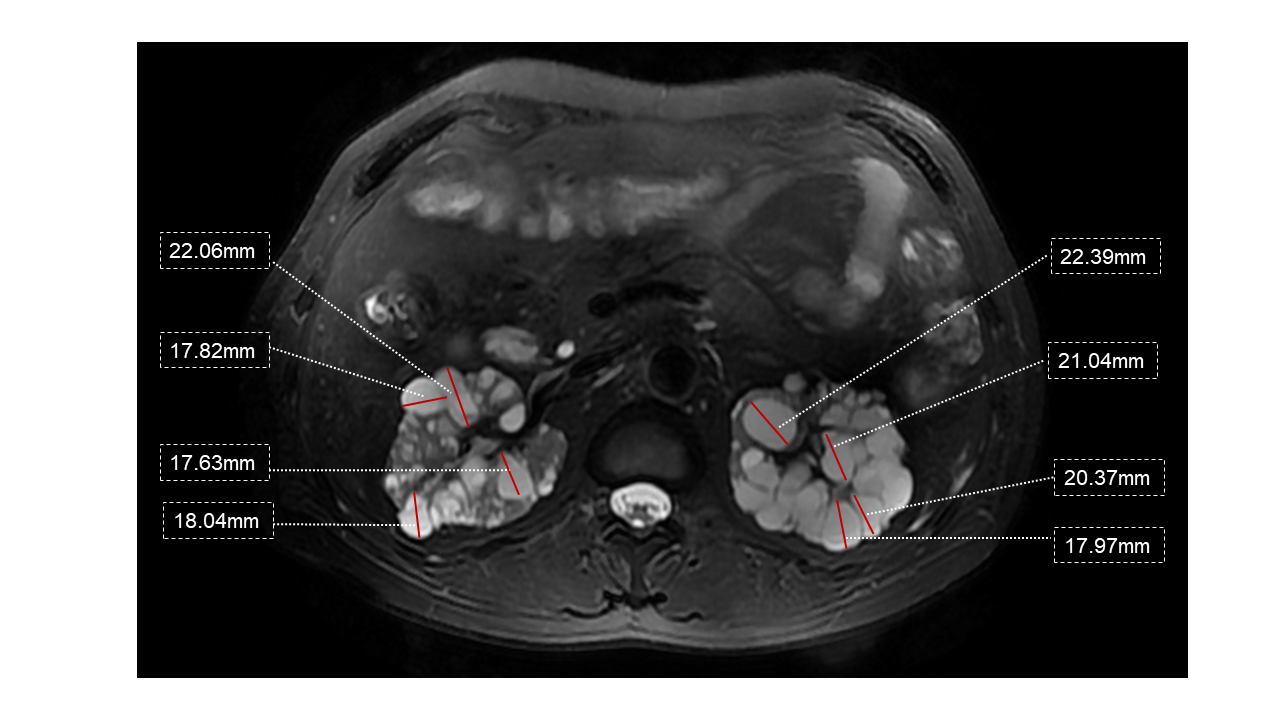


Renal MRI of the patient’s son (42 years old)

Supplement: Supplementary file 1 — Additional file 1. Ultrasound of the patient’s daughter (39 years old). Renal MRI of the patient’s son (42 years old). [file 12876_2020_1445_MOESM1_ESM.docx]
